# Spontaneous Topological States and Their Mutual Transformations in a Rare‐Earth Ferrimagnet

**DOI:** 10.1002/advs.202205574

**Published:** 2022-11-20

**Authors:** Shulan Zuo, Kaiming Qiao, Ying Zhang, Zhuolin Li, Tongyun Zhao, Chengbao Jiang, Baogen Shen

**Affiliations:** ^1^ Key Laboratory of Aerospace Materials and Performance (Ministry of Education) School of Materials Science and Engineering Beihang University Beijing 100191 P. R. China; ^2^ School of Materials Science and Engineering University of Science and Technology Beijing Beijing 100083 P. R. China; ^3^ Beijing National Laboratory for Condensed Matter Physics Institute of Physics Chinese Academy of Sciences Beijing 100190 P. R. China; ^4^ Songshan Lake Materials Laboratory Dongguan Guangdong 523808 P. R. China; ^5^ Ningbo Institute of Materials Technology & Engineering Chinese Academy of Sciences Ningbo Zhejiang 315201 P. R. China

**Keywords:** DyFe_11_Ti, ferrimagnets, magnetic anisotropy, spontaneous (bi‐)target bubbles, spontaneous magnetic skyrmions

## Abstract

Nontrivial chiral spin textures with nanometric sizes and novel characteristics (e.g., magnetic skyrmions) are promising for encoding information bits in future energy‐efficient and high‐density spintronic devices. Because of antiferromagnetic exchange coupling, skyrmions in ferrimagnetic materials exhibit many advantages in terms of size and efficient manipulation, which allow them to overcome the limitations of ferromagnetic skyrmions. Despite recent progress, ferrimagnetic skyrmions have been observed only in few films in the presence of external fields, while those in ferrimagnetic bulks remain elusive. This study reports on spontaneously generated zero‐field ground‐state magnetic skyrmions and their subsequent transformation into traditional magnetic bubbles via intermediate states of (bi‐)target bubbles during a magnetic anisotropy change in the rare‐earth ferrimagnetic crystal DyFe_11_Ti. Spontaneous reversible topological transformation driven by a temperature‐induced spin reorientation transition is directly distinguished using Lorentz transmission electron microscopy. The spontaneous generation of magnetic skyrmions and successive topological transformations in ferrimagnetic DyFe_11_Ti are expected to advance the design of topological spin textures with versatile properties and potential applications in rare‐earth magnets.

## Graphical abstract

Spontaneous magnetic biskyrmions and successive topological transformation into traditional magnetic bubbles via (bi‐)target bubbles are directly observed in a tetragonal ferrimagnetic crystal DyFe_11_Ti by changing the temperature using in situ Lorentz transmission electron microscopy. The spontaneous generation of easily interconverted topological states is a promising direction for designing and manipulating topological spin textures in ferrimagnetic crystals for future spintronic applications.

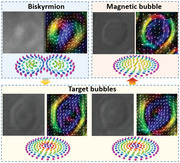

## 1 Introduction

Nanometre‐size particle‐like magnetic textures such as vortices,^[^ ^1^, ^2^ ^]^ (anti)skyrmions,^[^ ^3^, ^4^, ^5^ ^]^ merons,^[^ ^6^, ^7^ ^]^ bobbers,^[^ ^8^ ^]^ and bubbles^[^ ^9^, ^10^ ^]^ have attracted considerable attention in recent years because of their excellent topological properties, which are appropriate for future applications in spintronic devices such as racetrack memories. Since the first experimental discovery of magnetic skyrmions in ferromagnetic materials with a Dzyaloshinskii−Moriya interaction in 2009, significant efforts have been invested to tune the subtle competition of magnetic interactions to generate or manipulate skyrmions.^[^ ^3^ ^]^ Currently, skyrmion materials have been extended to diverse ferromagnets with different stabilization mechanisms.^[^ ^10^, ^11^, ^12^, ^13^, ^14^, ^15^, ^16^ ^]^ Skyrmions can be moved by an electric current, however, with a significant transverse deflection in response to an external electric current in ferromagnets.^[^ ^17^, ^18^, ^19^ ^]^ This is known as the skyrmion Hall effect and is caused by the Magnus force perpendicular to the applied current. Currently, this is a roadblock in most skyrmion‐based applications.^[^ ^20^, ^21^ ^]^ Theoretically, the skyrmion Hall effect can be avoided in antiferromagnetic materials with zero net magnetic moments because two equivalent but antiparallel magnetic sublattices cancel the transverse motion.^[^ ^22^, ^23^ ^]^ However, the experimental detection and study of noncollinear antiferromagnetic spin textures remain difficult.^[^ ^24^ ^]^ Instead, skyrmions in ferrimagnetic materials with partially compensated magnetic moments have been studied; they exhibit the advantages of antiferromagnetically coupled skyrmions.^[^ ^25^, ^26^, ^27^ ^]^ Therefore, it is crucial to unravel the topological magnetic textures in ferrimagnetic materials.

Although extensive studies have been conducted on ferromagnetic skyrmions in various crystals,^[^ ^3^, ^4^, ^12^, ^13^, ^28^, ^29^, ^30^ ^]^ experimental studies on ferrimagnetic skyrmions are limited to few films and multilayers^[^ ^25^, ^26^, ^27^, ^31^ ^]^ where the interfacial interactions play an important role; few ferrimagnetic crystals have been experimentally reported to host skyrmions. Further, both theoretical^[^ ^32^, ^33^, ^34^, ^35^ ^]^ and experimental^[^ ^36^, ^37^ ^]^ studies have proved the existence of spontaneous skyrmions in ferromagnets; however, this remains an open question for ferrimagnets. The underlying physics of the spontaneous topological spin‐texture stabilization involves a delicate balance of different micromagnetic energies wherein magnetic anisotropy is an important parameter.^[^ ^36^, ^37^ ^]^ The intrinsic topological spin texture and its stabilization mechanism might be extended to other materials, such as ferrimagnets, to further advance the specialties of these magnetic textures. In this case, the undesirable external magnetic field and the detrimental skyrmion Hall effect can be simultaneously avoided.^[^ ^24^, ^25^, ^26^, ^27^, ^38^ ^]^

In this study, spontaneous magnetic skyrmions and topological transformations between skyrmions and traditional magnetic bubbles via intermediate topology states called (bi‐)target bubbles were directly observed in the rare‐earth ferrimagnet DyFe_11_Ti using in situ Lorentz transmission electron microscopy (L‐TEM). The magnetic moments of Dy and Fe are inequivalent and antiparallel to each other, thereby forming a ferrimagnetic structure where the easy magnetization direction lies in the [110] axis within the basal (*a‐b*) plane at low temperatures and along the *c*‐axis at room temperature.^[^ ^39^, ^40^, ^41^ ^]^ This change in magnetic structure is caused by two successive spin reorientation transitions (SRTs): from the magnetic easy plane to the easy cone, and then to the *c*‐axis anisotropy with an increasing temperature due to the competitive contributions of the Dy and Fe sublattices to the magnetocrystalline anisotropy.^[^ ^39^, ^40^ ^]^ Biskyrmions are spontaneously generated in the region of easy‐cone anisotropy and replaced by (bi‐)target bubbles with vortex‐ring magnetic textures near the SRT between easy‐cone and easy‐axis anisotropy. This intermediate topology state is replaced by spontaneous magnetic bubbles due to the increased uniaxial anisotropy, which reveals the dominant role of magnetic anisotropy in stabilizing topological spin textures. The discovery of spontaneous skyrmions and successive topological transformations in the DyFe_11_Ti ferrimagnet provides an opportunity to explore various topological spin textures and stimulate further applications in rare‐earth ferrimagnetic crystals.

## 2 Results and Discussion

### 2.1 Basic Structure and Magnetic Properties of DyFe_11_Ti

**Figure 1:**
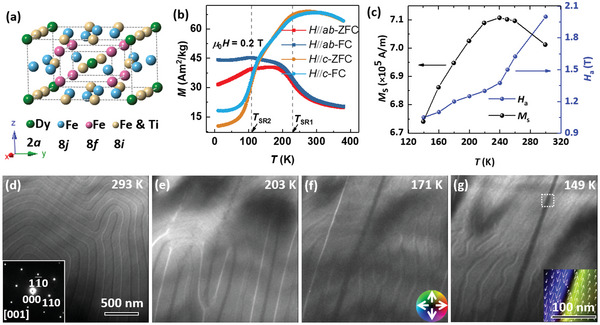
Structural and magnetic properties of ferrimagnetic DyFe_11_Ti alloys. a) Schematic of tetragonal ThMn_12_‐type structure (space group *I*4*/mmm*) DyFe_11_Ti with alternating stacks of Fe/Ti atomic layers and Dy‐Fe/Ti atomic layers along the *c* axis. b) Temperature‐dependent magnetization (*M*–*T*) for an oriented DyFe_11_Ti sample measured under a 0.2 T magnetic field applied parallelly (*H*//*c*) and perpendicularly (*H*//*ab*) to the *c* axis. c) Temperature‐dependent spontaneous magnetization *M*_s_ and effective anisotropy field *H*_a_. d–g) Typical underfocused L‐TEM images of the spontaneous magnetic domain structure evolution when cooling a DyFe_11_Ti thin plate near the [001] zone‐axis for the first time. d) Labyrinth domains with dominated *c*‐axis magnetization at 293 K, where the inset shows the selected‐area electron diffraction pattern along the [001] zone‐axis. e–g) Evolution of the magnetic domain structure as the direction of easy magnetization reorients away from the *c* axis and is close to the base plane during the SRT. The inset of (g) shows an in‐plane magnetic inductance mapping of 180° domains in the white boxed area obtained using the transport‐of‐intensity equation (TIE) analysis. The color wheel expounding the magnitude and direction of the in‐plane magnetic inductance represented by the color and white arrows is presented in the inset of (f), where the dark color indicates the upward (downward) magnetizations. The scale bar in the inset is 100 nm.

Rare‐earth ferrimagnetic DyFe_11_Ti with a high Curie temperature (*T*_C_ ≈ 550 K) crystallizes in a centrosymmetric tetragonal ThMn_12_‐type structure (space group *I*4*/mmm*)^[^ ^39^, ^42^ ^]^ where Dy atoms occupy the 2a site, whereas the transition metal atoms preferentially occupy the three inequivalent 8i, 8j, and 8f atomic sites, as shown in **Figure** **1**a. The as‐prepared sample shows a single‐phase tetragonal ThMn_12_‐type structure with crystal parameters of *a* = *b* = 8.506(9) Å and *c* = 4.789(7) Å at room temperature (Figure S1a, Supporting Information). In this alloy, the effective magnetic anisotropy fields of the Fe and Dy sublattices prefer orientation moments along the *c*‐axis and basal plane, respectively.^[^ ^39^, ^42^ ^]^ Thus, the temperature‐driven SRT from easy‐axis to easy‐cone at ≈230 K (*T*_SR1_) is followed by another SRT from easy‐cone to easy‐plane at ≈108 K (*T*_SR2_) (Figure 1b and Figure S1c, Supporting Information). The ThMn_12_‐type crystal structure is well retained despite the large change in the magnetic anisotropy (Figures S1b and S2, Supporting Information). The temperature‐dependent spontaneous magnetization *M*_s_ (Figure 1c), calculated by fitting the magnetization curves (Figure S1d, Supporting Information) based on the law of approaching saturation, exhibits a maximum value near *T*_SR1_; this is due to the different Dy and Fe magnetic moment changes with temperature.^[^ ^40^ ^]^

The magnetic domain structure evolution during the SRT is revealed by in‐situ L‐TEM imaging while cooling a DyFe_11_Ti specimen along the [001] orientation (Figure 1d–g). Typical labyrinth domains are observed at room temperature (293 K, Figure 1d) because of the *c*‐axis magnetic anisotropy dominated by the Fe sublattice (Figure S2a, Supporting Information). Stripe domains develop (Figure 1e) as the direction of the easy magnetization rotates gradually away from the *c* axis when the temperature is lowered below *T*_SR1_ (Figure S2e–h, Supporting Information). Thus, the labyrinth domains are replaced by stripes inside the microsize 180° domains (Figure 1f) under the easy‐cone magnetic anisotropy. These stripes gradually vanish when the temperature is lowered further because of the enhanced easy‐plane anisotropy (Figure S2i–k, Supporting Information); the residual stripe domains at 149 K show a worm‐like contrast as presented in the lower‐left part of Figure 1g, where the 180° domain walls are approximately parallel to the [1$\bar{1}$0] direction coinciding with the easy magnetization direction of [110] axis in the basal plane at low temperature.^[^ ^40^, ^41^ ^]^ The overall magnetic domain evolution corresponds well with the anisotropy change caused by the SRT. Therefore, the gradual decrease in the perpendicular magnetocrystalline anisotropy (Figure 1c) suggests that the initial labyrinth domains at room temperature transfer into conventional 180° domains at low temperatures with the appearance of stripe domains during the first cooling process.

### 2.2 Spontaneous Topological States and Transformation without External Fields

**Figure 2:**
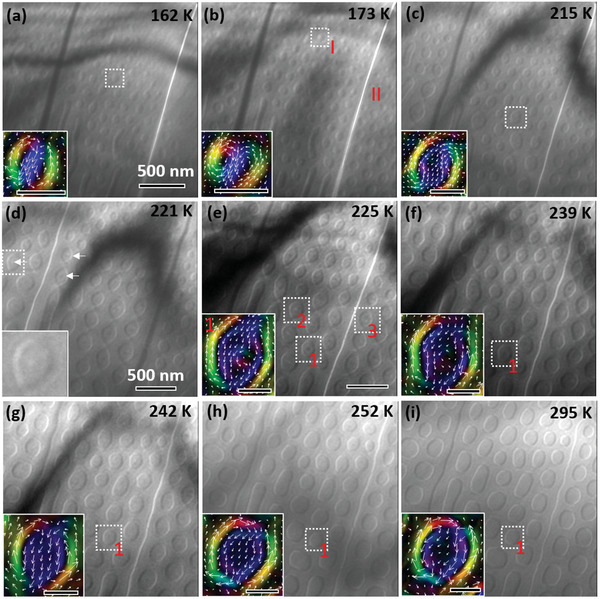
L‐TEM images of the magnetic domain evolution with increasing temperature at zero fields. a) Spontaneous biskyrmions develop inside micrometer‐sized 180° domains. b) The biskyrmions gradually increase with an increasing temperature. The insets in (a) and (b) present the spin configurations, obtained via TIE analyses, of the biskyrmions. c) The biskyrmions become larger; the inset shows the spin configuration of an enlarged single biskyrmion. d) Weak vortex‐like cores appear in some nanodomains; the inset shows a boxed nanodomain with a weak vortex‐like core. e) Magnetic configurations host obvious vortex‐like cores with different contrasts, where the white boxes with red numbers mark three typical cases. f,g) The dot contrast evolves with temperature and vanishes gradually with a further increase in temperature. The insets in (e)–(g) show the in‐plane magnetic inductance distributions of boxed nanodomains at different temperatures obtained via the TIE analyses. h) Spontaneous magnetic bubbles dismissing dot contrasts above *T*_SR1_ (230 K). i) The spontaneous magnetic bubbles are stable even at 295 K. The insets in (h) and (i) show spin textures of magnetic bubbles at the corresponding temperatures. The scale bars in the insets are 100 nm. All L‐TEM images are under focused except (d), which is overfocused.

**Figure 3:**
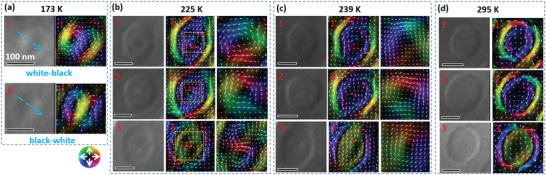
Spin configurations of different topological states. a) Biskyrmions with opposite chirality distribution and the corresponding magnetic textures at 173 K obtained using the TIE analysis. b) (Bi‐)target bubbles with vortex‐like cores in the intermediate state at 225 K. c) Target bubbles with weakened vortex‐like cores. d) Traditional type‐II bubbles.

Topological spin textures are preferentially generated during successive heating processes in comparison with the irreversible first cooling process. When the temperature is increased from 149 to 162 K, nanodomains with half‐white and half‐dark contrast spontaneously emerge (**Figure** **2**a) and are identified as magnetic biskyrmions^[^ ^13^ ^]^ by combining the transport‐of‐intensity equation (TIE) analyses (inset of Figure 2a). The biskyrmions become denser with increasing temperature (Figure 2b) and then remain unchanged within a certain temperature range. Notice that biskyrmions with opposite chirality distributions are separated by a 180° domain wall (Figures 2b and **3**a). The biskyrmions enlarge (Figure 2c) and then, with a further increase in temperature, develop into nanodomains with vortex‐like cores hosting dot contrasts (indicated by white arrows in Figure 2d). These cores become clearer with a slight increase in temperature (Figure 2e), and they show different contrasts including black, white, and white‐black pairs, which will be analyzed later. With a further increase in temperature, these dots gradually vanish (Figure 2f,g), and the corresponding nanodomains evolve into magnetic bubbles (Figure 2h) with the same chirality as the initial biskyrmions. Spontaneous magnetic bubbles are stable even after being heated to room temperature (Figure 2i), in contrast to the initial labyrinth domains (Figure 1d). Once spontaneous magnetic bubbles form, the transformation between the magnetic bubbles and biskyrmions via the intermediate state with vortex‐like cores occur in a reproducible and reversible manner as the temperature changes (Figure S3, Supporting Information). This is in contrast to the evolution of labyrinth domains in the first cooling process (Figure 1d–g).

Next, we focused on the detailed spin textures in the nanodomains in Figure 3, which are extracted from Figure 2. The magnetic textures of biskyrmions with opposite chirality distributions (marked as I and II) and separated by a 180° wall (Figure 2b) are presented in Figure 3a. In contrast to the half‐dark and half‐white contrast of the biskyrmions, the nanodomains with thin cylindrical domain walls separating them from the outside opposite magnetization are traditional magnetic bubbles (Figure 3d).^[^ ^43^, ^44^ ^]^ Bloch lines appear in the walls of these bubbles, which indicate type II magnetic bubbles.^[^ ^43^, ^44^ ^]^ Thus, the nanodomains with vortex‐like cores evolving from the enlarged biskyrmions are identified as the intermediate states (Figure 3b,c) between the biskyrmions and magnetic bubbles. In this intermediate state, magnetic whirling configurations resemble magnetic bubbles but with weak cores, wherein some bubbles hosting a single core (marked as “1” and “2”) are named “target bubbles” following the previous convention^[^ ^45^, ^46^ ^]^ while the bubbles with double cores possessing opposite chirality (marked as “3”) are renamed “bi‐target bubbles.” A whirling spin texture with dot magnetic contrast has two internal degrees of freedom,^[^ ^1^, ^2^, ^45^, ^46^ ^]^ i.e., polarity *p* is defined as the direction of the spins at the core, and the circulation *c* (i.e. chirality or helicity) indicates the rotational direction of the in‐plane magnetizations along the perimeter (i.e., clockwise or counterclockwise).

As shown in Figures 2e and 3b, inner cores with different contrasts of dark, white, or dark‐white pairs randomly distributed in the (bi‐)target bubbles correspond to the different chirality of the cores in correlation with the helicity freedom in centrosymmetric materials,^[^ ^12^, ^13^ ^]^ whereas the outer ring inherits the chirality of the corresponding biskyrmions at low temperatures. The in‐plane magnetic inductance at the core position is much weaker than the outer domain walls in (bi‐)target bubbles, which can be better identified in the corresponding phase maps (Figure S4, Supporting Information). This difference is closely related to the 3‐dimensional magnetic moment distribution in the (bi‐)target bubbles throughout the thickness of the sample,^[^ ^8^, ^45^ ^]^ and we leave it for future study. The dedicated spin textures of the (bi‐)target bubbles evolve with temperature, where the core contrast becomes weaker with increasing temperature, and one of the dots in the bi‐target bubble “3” vanishes under stronger perpendicular magnetic anisotropy (Figure 3c). These inner cores almost completely vanish into dominant perpendicular magnetization (Figure 3d) as the temperature increases away from *T*_SR1_ (230 K). Therefore, the biskyrmions evolve into traditional type‐II magnetic bubbles under gradually enhanced perpendicular anisotropy with the appearance of the intermediate state of (bi‐)target bubbles, which indicates a smooth evolution of magnetic textures. During this process, vortex‐like whirling spin textures with dot contrasts become the cores via rotating the magnetic moments and eventually transform into uniform perpendicular magnetization under stronger perpendicular magnetic anisotropy, resulting in the formation of magnetic bubbles.

The (bi‐)target bubble has a pair of Bloch lines in the outer ring, and therefore, the topological charge is determined by the internal structure or configuration of the Bloch lines.^[^ ^9^ ^]^ According to the definition of topological charge, the target bubble with a single core has a topological charge of 1,^[^ ^9^ ^]^ whereas the bi‐target bubble with two cores has a topological charge of 2. Traditional type II magnetic bubbles have a topological charge of 0. Thus, the topological charge changes from 2 to 1 and then to 0 during the topological transformation from biskyrmions to (bi‐)target bubbles, and then to traditional type II magnetic bubbles. During this evolution, the outer rings of the (bi‐)target bubble and type II magnetic bubbles evolve from the periphery of enlarged biskyrmions, and Bloch lines are formed by the overlapping regions of the outer parts of the constituent skyrmions in the biskyrmion.^[^ ^47^ ^]^

**Figure 4:**
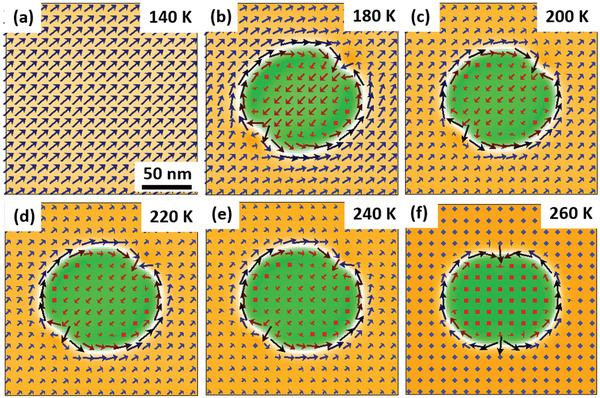
Simulated magnetic texture evolution from the biskyrmion to the bubble at zero fields in DyFe_11_Ti. a) Single large domain. b) Biskyrmion with a topological number of 2, which is integrated over the *x* and *y* directions of the simulation image. c–f) The in‐plane magnetization component of the spin configurations gradually decreases with an increase in temperature, which indicates the gradual transition from the biskyrmion to a magnetic bubble. Magnetic parameters at different temperatures were selected based on experimental values. The scale bar is 50 nm. The out‐of‐plane magnetization is depicted by the regions in yellow (+m_z_) and green (−m_z_), while the white regions with black arrows represent the in‐plane magnetization.

The SRT‐driven magnetic texture transformation can be well demonstrated using micromagnetic simulations based on the magnetic parameters of DyFe_11_Ti (see Experimental Section). A large single domain is observed first (**Figure** **4**a), and the biskyrmion spin texture is spontaneously generated (Figure 4b) as the effective anisotropy field *H*_a_ gradually increases and the angle between the effective magnetic anisotropy field and *c* axis decreases based on experimental results at specific temperatures (see Table S1 for more details). When continuing to adjust the parameters in the same way as the SRT from easy cone to easy *c*‐axis anisotropy, the vertical magnetization component in the core of the simulated nanodomain increases gradually (Figure 4c–e) and becomes dominant (Figure 4f), which indicates a transition into a magnetic bubble. The two cores of the simulated biskyrmions (Figure 4b) are clearly distinguished from the dominated perpendicular magnetization of the magnetic bubble (Figure 4f). The biskyrmion core shows vertical net magnetization that is opposite to the magnetization surrounding the biskyrmion. The simulated biskyrmion also shows a vertical net magnetization at the core, and thus in‐plane magnetization of cores seems to have a magnitude weaker than the surrounding domain walls. This transition cannot occur if only *H*_a_ or *M*_s_ is increased (Figure S5, Supporting Information) within experiment values during the SRT, but happens solely by changing the easy magnetization axis (Figure S6, Supporting Information), which indicates the key role of the easy magnetization direction. Notice that the size of the biskyrmions clearly changes when varying *H*_a_ or *M*_s_ (Figure S5, Supporting Information). These facts suggest that the experimental observation of spontaneous topological transformations can be mainly attributed to the subtle competition between the dipolar interaction and magnetic anisotropy during the SRT. Below *T*_SR1_, the easy‐cone anisotropy based on the competition between the in‐plane and out‐of‐plane magnetization could give rise to the formation of spontaneous biskyrmions under certain dipolar interaction energies. Above *T*_SR1_, the formation of spontaneous bubble is promoted. The (bi‐)target bubbles in DyFe_11_Ti exist only within a narrow temperature range around *T*_SR1_ (Figure 2), which implies a strict stability condition. The divergence in the simulation results is caused by the simple model and inaccurate parameters.

### 2.3 Magnetic Texture Evolution under External Magnetic Fields in Connection with Polarity

**Figure 5:**
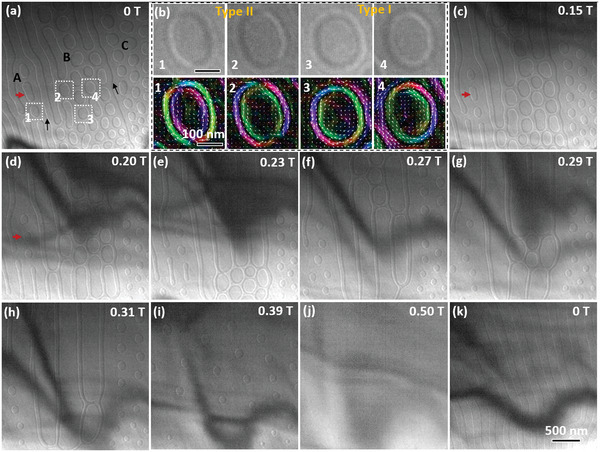
Magnetic bubble evolution under perpendicular magnetic fields at room temperature (293 K). a) Spontaneous magnetic bubbles at room temperature. The black arrows indicate two large domain walls that divide the image into three parts marked “A,” “B,” and “C.” b) Type I and Type II bubbles with opposite chirality numbered in (a); the scale bars are 100 nm. c,d) Dumbbell‐like domains gradually evolve into magnetic bubbles in “A” and “C,” whereas magnetic bubbles in “B” become significantly large with increased magnetic fields. e–g) Labyrinth domains in “A” and “C” transform into magnetic bubbles while magnetic bubbles in “B” appear randomly. h) Magnetic bubbles in “B” annihilate into labyrinth domains. i) Complete bubble state. j) Saturation state with uniform magnetic contrast. k) Labyrinth domains at residual state after removing the magnetic field. The scale bar for large images is 500 nm.

The polarity of magnetic bubbles cannot be measured directly via L‐TEM; however, it can be deduced from their magnetic field‐driven evolution.^[^ ^46^ ^]^ Spontaneous magnetic bubbles are stable at room temperature because of the cooling (Figure 1d–g) and successive heating (Figure 2) processes; however, some type II bubbles evolve into type I bubbles being put at room temperature for a long time (**Figure** **5**a,b). Skyrmion bubbles with continuous magnetization in the cylindrical wall and a topological charge of 1 are obtained via diminishing Bloch lines.

We also investigate the stability and dynamic behavior of these spontaneous magnetic textures under perpendicular magnetic fields at room temperature (Figure 5). Magnetic bubbles separated by the 180° walls (indicated by black arrows in Figure 5a) evolve differently under magnetic fields, wherein two large domain walls divide the image of Figure 5a into three parts marked as “A,” “B,” and “C.” An increase in the applied perpendicular magnetic field shrinks the broken labyrinth domains into bubbles in “A” and “C” (Figure 5c,d) and enlarges significantly the magnetic bubbles in “B,” which makes some of them evolve into dumbbell‐like domains (Figure 5d). The residual magnetic bubbles in “B” become unstable and move randomly (Figure 5e–g) before totally transforming into labyrinth domains (Figure 5h) that evolve into magnetic bubbles when the magnetic field is increased further. Simultaneously, labyrinth domains in “A” and “C” evolve into magnetic bubbles with increasing magnetic fields (Figure 5e–g); this different evolution is related to the magnetization at the bubble cores in “A”/“C” and “B” because the magnetic bubble is stable only if the applied external magnetic field is antiparallel to the magnetization direction of its core.^[^ ^46^ ^]^ This indicates the opposite polarity of magnetic bubbles on either side of a large 180° domain wall, which is similar to the chirality (Figures 2 and 3). The magnetic bubbles in “A” and “C” behave similarly because of the same polarity. Since the chirality and polarity of the type‐II bubbles are inherited from the biskyrmions, we infer that the biskyrmions and (bi‐)target bubbles located on either side of the large domain walls also host opposite polarities. A complete bubble state with uniform polarity forms at ≈0.39 T (Figure 5i) and vanishes into the saturated state at 0.50 T (Figure 5j). After the removal of the magnetic field, the residual state becomes labyrinth domains (Figure 5k) instead of spontaneous magnetic bubbles (Figure 5a). These zero‐field labyrinth domains could transform into magnetic bubbles with a lower density because each stripe domain apparently shrinks to a type‐II bubble with increasing magnetic fields (Figure S7, Supporting Information). In the DyFe_11_Ti, the labyrinth‐like domains are the ground state at room temperature; however, the bubble state has been checked with good stability at room temperature (Figure S8, Supporting Information).

## 3 Conclusions

The emergence of spontaneous topological states including magnetic skyrmions, (bi‐)target bubbles, and conventional bubbles is clearly demonstrated in the ferrimagnetic crystal DyFe_11_Ti using in situ L‐TEM. Spontaneous reversible topological transitions between biskyrmions and conventional bubbles occur successively via an intermediate state of (bi‐)target bubbles with inherited chirality and polarity as changing the temperature. The spontaneous generation of easily interconverted topological states points to a promising direction for the design and manipulation of topological spin textures. Our findings shed light on discovering unknown, spontaneous topological spin textures and their mutual transitions in ferrimagnetic crystals, and on additional possibilities to exploit new materials with topological states for future spintronic applications.

## 4 Experimental Section

### Sample Preparation

A polycrystalline tetragonal DyFe_11_Ti alloy with a nominal composition was prepared by arc melting mixtures of highly pure Dy, Fe, and Ti metals under a high‐purity argon atmosphere. Rare‐earth Dy (3 wt%) in excess over the stoichiometric composition was added during arc melting to compensate for weight loss. The as‐obtained ingot was sealed in a quartz tube under a high‐purity argon atmosphere, annealed at 1223 K for 72 h, and then quenched in liquid nitrogen to ensure homogeneity. The prepared samples were analyzed using powder X‐ray diffraction (Rigaku Smartlab diffractometer with Cu *K*_*α*_ radiation), and lattice constants were calculated via Rietveld refinements of the X‐ray diffraction patterns.

### Magnetic Measurements

Magnetic property measurements of the sample were performed using a superconducting quantum interference device–vibrating sample magnetometer. The spontaneous magnetization *M*_s_ was obtained by fitting the magnetization curves at magnetic fields higher than 2.0 T for a polycrystalline sample based on the approach to the saturation law. The singular point detection method^[^ ^48^ ^]^ was employed to calculate the effective magnetocrystalline anisotropy field *H*_a_ of an oriented polycrystalline DyFe_11_Ti sample prepared by aligning milled powders with epoxy resin under a magnetic field of 3 T at room temperature.

### L‐TEM Measurements

The polycrystalline ingot was cut into slices thinned by mechanical polishing, dimpling, and argon ion milling for the preparation of the TEM sample. The magnetic domain structures were observed using the Fresnel method in a JEOL‐dedicated L‐TEM (JEOL 2100F) with a negligible remnant magnetic field around the sample. In this technique, magnetic domain walls were imaged as bright or dark contrast on the defocused (under‐ or over‐focused) image planes because of the deflected electron beam caused by the Lorentz force. A liquid‐nitrogen TEM sample holder with a nominal temperature range of 120–300 K was used to observe the magnetic domain evolution with temperature. The defocus images were taken under defocus values of ≈250 µm above 250 K, ≈280 µm between 242 and 203 K, and ≈400 µm below 200 K, which correspond to spatial resolutions of ≈24, 24, and 28 nm, respectively.^[^ ^49^, ^50^ ^]^ A conventional L‐TEM (JEOL 2100F) was used to apply a perpendicular magnetic field by gradually increasing the objective lens current. The specimen was not tilted at any point during imaging, even at different temperatures and applied magnetic fields. Detailed spin textures were obtained by analyzing the corresponding L‐TEM images (under‐, over‐, and just‐focus images) using the commercial QPt software based on TIE.^[^ ^51^ ^]^ The colors and arrows in the final in‐plane magnetic inductance maps depict the magnitude and orientation of the in‐plane inductance distribution whereas the dark color denotes the out‐of‐plane magnetization. The thickness of the L‐TEM specimen was measured using the electron energy loss spectrum in TEM and was ≈100 nm (Figure S9, Supporting Information).

### Micromagnetic Simulation

Micromagnetic simulations were performed using the 3D object‐oriented micromagnetic framework (OOMMF) code based on the Landau–Lifshitz–Gilbert equation^[^ ^52^ ^]^ to study the magnetic structure evolution with magnetic parameters such as magnetic anisotropy (*H*_a_) and saturated magnetization (*M*_s_) at a fixed exchange constant (*A* = 1.0 × 10^−11^ J m^−1^). The exchange constant *A* was estimated using *δ* = $\pi \sqrt{A/K_{u}}$, where *δ* represents the domain wall width and was ≈11.5 nm at room temperature, based on the L‐TEM results. A thin plate of 200×200×20 nm^3^ with periodic boundary conditions and rectangular mesh sizes of 2×2×2 nm^3^ was used to run the program. In this simulation, demagnetization was performed using a common method. The skyrmion number integrated over the *x*‐ and *y*‐directions of the simulation image was defined as *N*_s_ = $\frac{1}{4\pi }\int \;\;\;\int n\cdot \left(\frac{\partial n}{\partial x}\times \frac{\partial n}{\partial y}\right)dxdy$, where ***n*** represents the direction vector of magnetization.^[^ ^17^ ^]^

## Conflict of Interest

The authors declare no conflict of interest.

## Supporting information

Supporting Information

## Data Availability

The data that support the findings of this study are available from the corresponding author upon reasonable request.
