# Supplementary material for: Spontaneous Topological States and Their Mutual Transformations in a Rare‐Earth Ferrimagnet
Source: Adv Sci (Weinh). 2022 Nov 20;10(3):2205574. doi: 10.1002/advs.202205574 (PMC9875609; doi:10.1002/advs.202205574)
Supplement: Supplementary file 1 — Supporting Information [file ADVS-10-2205574-s001.pdf]

ADVANCED SCIENCE
Open Access

Supporting Information

for *Adv. Sci.*, DOI 10.1002/advs.202205574

Spontaneous Topological States and Their Mutual Transformations in a Rare-Earth Ferrimagnet

Shulan Zuo, Kaiming Qiao, Ying Zhang*, Zhuolin Li, Tongyun Zhao, Chengbao Jiang and Baogen Shen*

Supporting Information

Spontaneous Topological States and Their Mutual Transformations in a Rare-Earth Ferrimagnet

Shulan Zuo, Kaiming Qiao, Ying Zhang,* Zhuolin Li, Tongyun Zhao, Chengbao Jiang, and Baogen Shen*

S1. Basic structure and magnetic properties of $DyFe_{11}Ti$

The powder X-ray diffraction (XRD) pattern of $DyFe_{11}Ti$ and its Rietveld refinement at room temperature indicate a single-phase tetragonal $ThMn_{12}$-type structure (space group *I4/mmm*) (Figure S1a). All peaks in the XRD patterns at various temperatures can be indexed using this $ThMn_{12}$-type structure, which demonstrate the absence of structural phase transitions at different temperatures (Figure S1b).

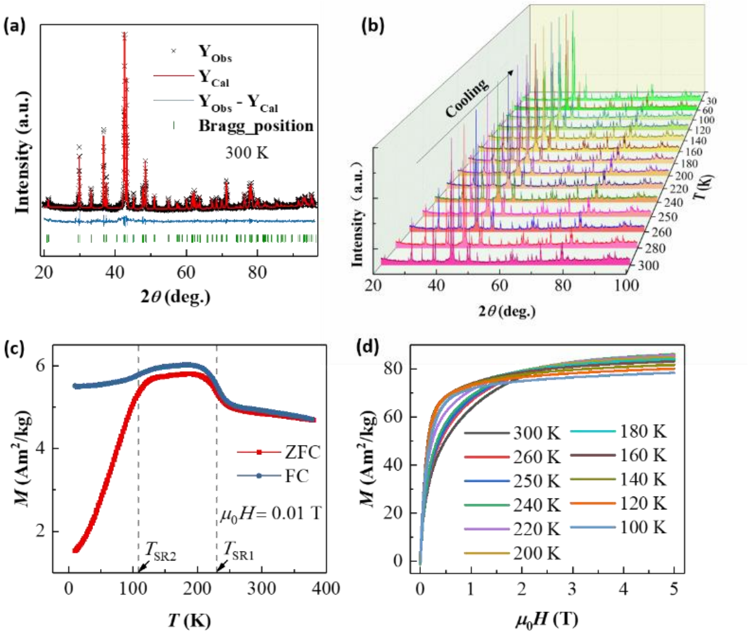

Figure S1. (a) Rietveld refinement of powder X-ray diffraction (XRD) pattern at room temperature. (b) XRD patterns at various temperatures. (c) Temperature-dependent magnetisation (*M*–*T*) of a polycrystalline $DyFe_{11}Ti$ sample measured under zero-field cooling (ZFC) and field-cooling (FC) models at a magnetic field of 0.01 T. (d) Magnetic-field-dependent magnetisation (*M*–*H*) at different temperatures.

The clear changes in the temperature-dependent magnetisation ((*M*–*T*) curves in Figure S1c) of bulk $DyFe_{11}Ti$ at approximately 230 (T_{SR1}) and 108 K (T_{SR2}) correspond to two spin reorientation transition (SRT) temperatures. The magnetic-field-dependent magnetisation (*M*–

H) of the $DyFe_{11}Ti$ sample (Figure S1d) changes slightly with temperature. The spontaneous magnetisation M_s, calculated by fitting the high-field portions of these *M*–*H* curves (≥ 2.0 T) according to the law of approach to saturation, exhibits a non-monotonic variation (Figure 1c).

S2. Calculation of effective anisotropy field H_a

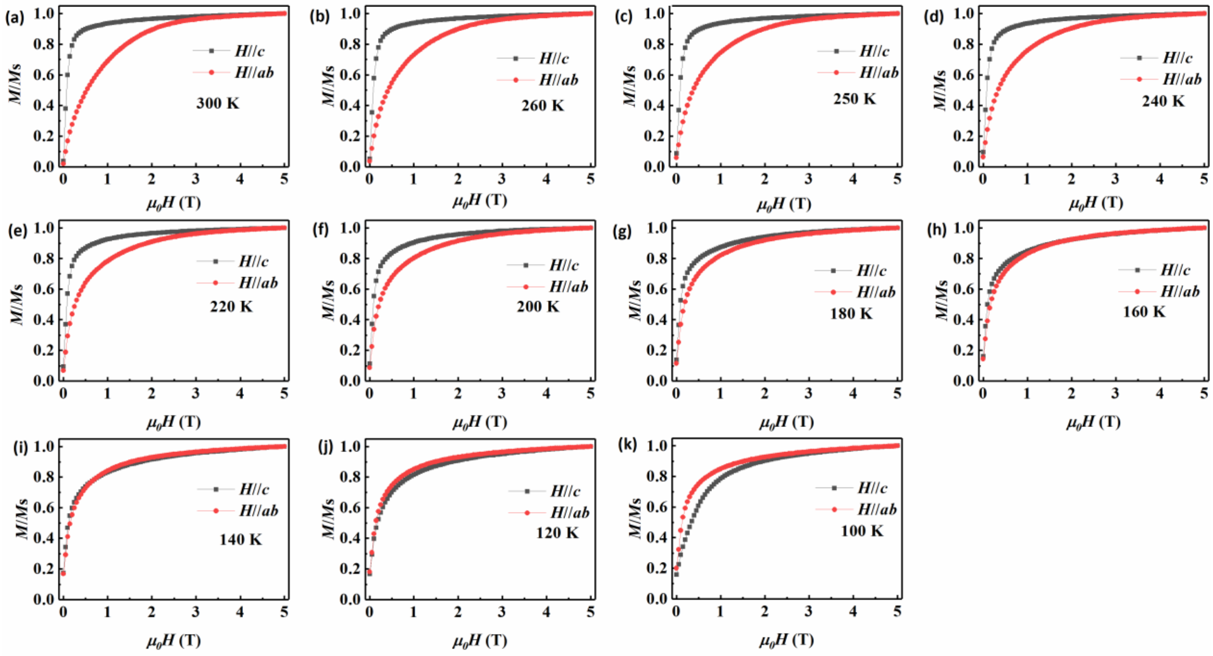

Figure S2. *M*–*H* curves of an oriented sample at various temperatures measured by applying magnetic fields parallelly (*H*//c) and perpendicularly (*H*//*ab*) to the *c* axis.

The *M*–*H* curves for an oriented sample are measured at different temperatures while applying the magnetic field perpendicularly (*H*//ab) and parallelly (*H*//c) to the *c* axis to characterise the change in magnetic anisotropy (Figure S2). The oriented sample, which possesses a strong crystallographic *c*-axis orientation, is prepared at room temperature by aligning milled powders with epoxy resin at a magnetic field of 3 T. The *M*–*H* curves between 300 and 240 K (Figure S2a–d) confirm that the *c*-axis is the direction of easy magnetisation. The difference between the magnetisation curves of *H*//ab and *H*//c decreases with a decrease in the temperature below T_{SR1} (Figure S2e–i). Eventually, the direction of easy magnetisation lies in the *ab* plane when reducing the temperature below T_{SR2} (Figure S2j,k).

The effective anisotropy field between 300 and 140 K is calculated using the singular point detection technique,[1,2] and it slightly decreases with decreasing temperature, as shown in Figure 1c; in this figure, the *M*–*H* curves of *H*//*ab* (red lines in Figure S2a–i) are used for the calculation.

S3. Spontaneous magnetic bubble evolution in the second cooling process

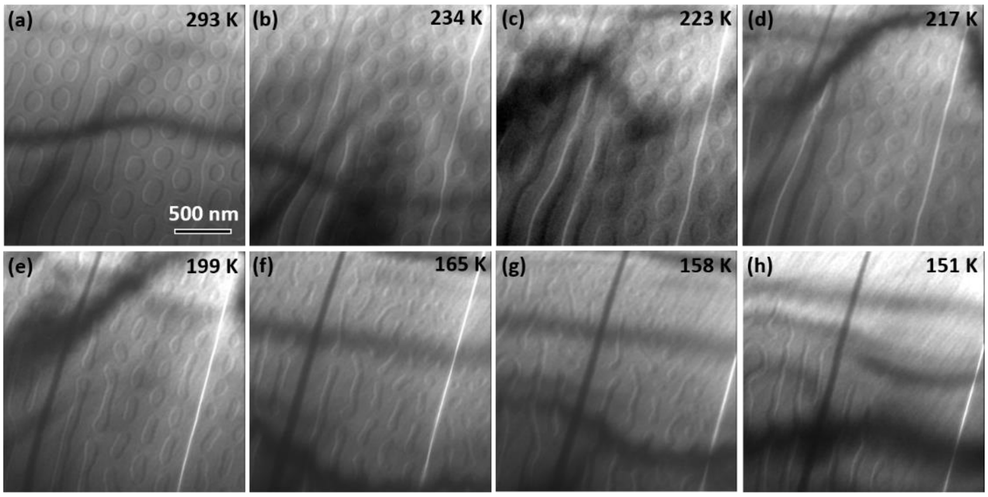

Figure S3. Under-focused L-TEM images of magnetic bubble evolution with decreasing temperature at zero magnetic fields. (a) Spontaneous magnetic bubbles at room temperature (293 K). (b) (Bi-)target bubbles with weak vortex-like cores. (c,d) Cores with different magnetic contrast become clearer. (e–h) Nanodomains and broken stripes evolve first into biskyrmions and then vanish gradually.

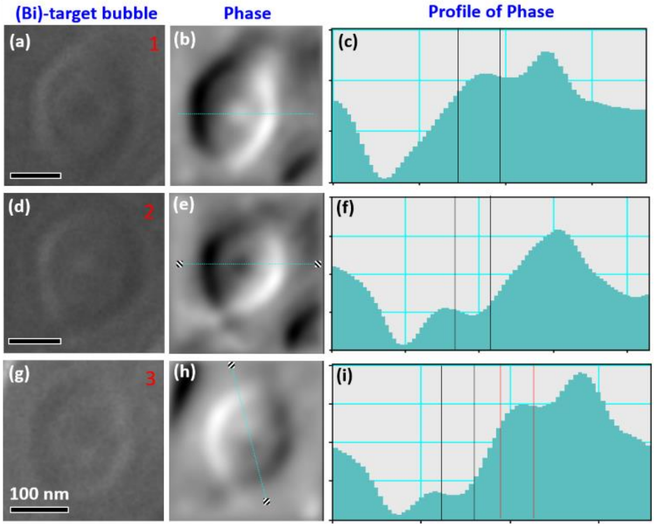

Figure S4. Under focused L-TEM images of (a,d) target bubbles, (g) bi-target bubble, and (b,e,h) corresponding phase maps. (c,f,i) Phase line profiles across the domain marked by the dotted green line in (b,e,h), where black and red vertical lines mark the location of the cores.

The spontaneous magnetic bubbles slightly distort at low temperatures near T_{SR1}, and they evolve into (bi-)target bubbles (Figure S3a,b). These (bi-)target bubbles become smaller (Figure S3c,d) with a further decrease in temperature, and they transform into biskyrmions with the disappearance of the weak vortex-like cores (Figure S3e,f). Biskyrmions gradually vanish into large in-plane domains with decreasing temperature because of the weakened perpendicular magnetic anisotropy during the SRT from the easy cone to the easy plane

(Figure S3g,h). The core position in the (bi-)target bubbles can be identified using the corresponding phase maps (Figure S4). It is demonstrated that the in-plane magnetic inductance at the core position is weaker than that at the outer domain walls in the (bi-)target bubbles.

S4. Micromagnetic simulation of magnetisation states in $DyFe_{11}Ti$

Table S1. Detailed magnetic parameters for micromagnetic simulations at different temperatures where the axis presents the direction of H_a.

T (K)	140	180	200	220	240	260
H_a (×10^5 A/m)	7	7	10.0	10.3	10.9	12.9
M_s (×10^5 A/m)	6.7	6.9	7.0	7.1	7.1	7.1
axis	[1 1 4]	[1 1 6]	[1 1 7]	[1 1 8]	[1 1 10]	[0 0 1]

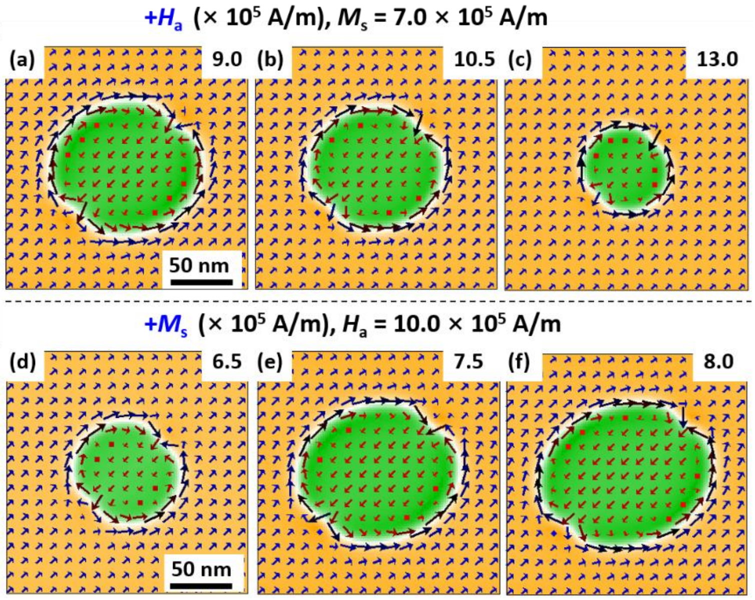

Figure S5. (a–c) Simulated biskyrmion evolution as a function of H_a at fixed M_s. (d–f) Simulated biskyrmion evolution as a function of M_s at fixed H_a. No clear change occurs in the detailed spin textures of the nanodomain except in the size.

Magnetic parameters such as the effective anisotropy field H_a and saturation magnetisation M_s of $DyFe_{11}Ti$ at specific temperatures (Figure 1c) are used to perform micromagnetic simulations to understand the magnetic texture evolution with temperature, as shown in Table S1 wherein the axis is selected based on the neutron diffraction result,[3] which presents the change in the direction of easy magnetisation during the SRT. The effective anisotropy field H_a at 180 K used to simulate the biskyrmion is slightly smaller than the experimental value. The overall domain structure is summarised in Figure 4 of the main

text. The magnetic domain structure evolution is studied by changing one magnetic parameter at 200 K to demonstrate the role of different factors in promoting topological transitions during the SRT, and it is shown that the topological spin texture hardly changes with the variation in H_a or M_s (Figure S5) within the experimental values during the SRT except for the biskyrmion size. Conversely, the transition from the biskyrmion to the bubble occurs (Figure S6) while solely changing the direction of H_a.

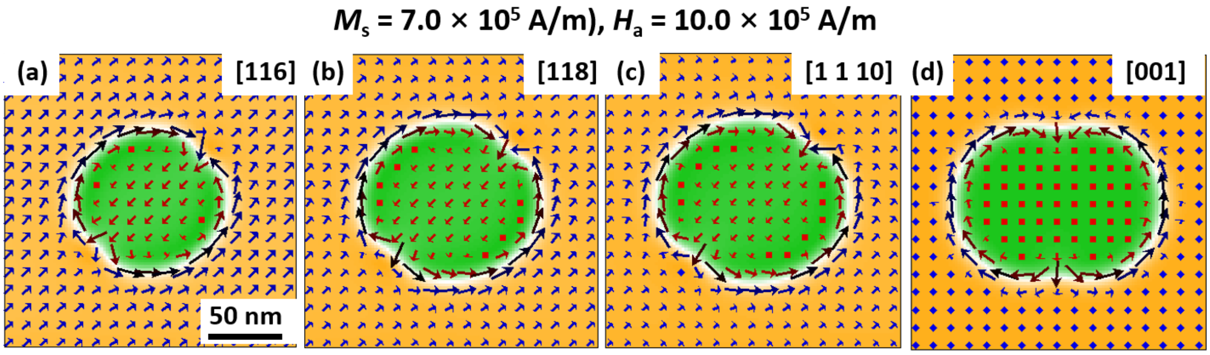

Figure S6. Simulated biskyrmion evolution with a decreasing angle between H_a and c axis while fixing H_a and M_s. As the easily magnetised axis gets closer to the c-axis, the vertical magnetisation component of the nanodomain increases gradually.

S5. Magnetic field-driven labyrinth domain evolution at room temperature

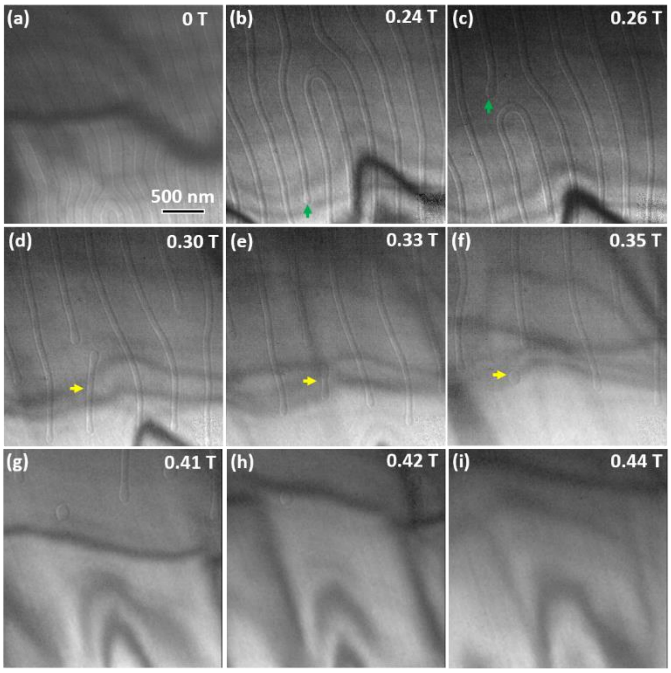

Figure S7. Labyrinth-domain evolution under perpendicular magnetic fields at room temperature (293 K). (a) Labyrinth domains at zero field. (b,c) Labyrinth domains gradually shrink with increasing magnetic fields. (d–f) Broken stripe gradually evolves into the magnetic bubble as indicated by the yellow arrow. (g)

Mixed state of magnetic bubbles and stripes. (h) Almost complete bubble state. (i) Saturation state with a uniform magnetic contrast.

Figure S7 shows the labyrinth domain evolution under a perpendicular magnetic field in $DyFe_{11}Ti$ at room temperature. The labyrinth domains (Figure S7a) shrink under a perpendicular magnetic field (indicated by a green arrow in Figures S7b,c) and become stripe-like domains. The shrunken stripe gradually evolves into a type-II bubble with increasing magnetic field (indicated by yellow arrows in Figures S7d–f). The bubbles show a low density even though the stripes continue to translate into magnetic bubbles before vanishing (Figures S7g–i) because stripes are rarely broken in this evolution, in contrast with previously reported magnetic bubble materials.[4]

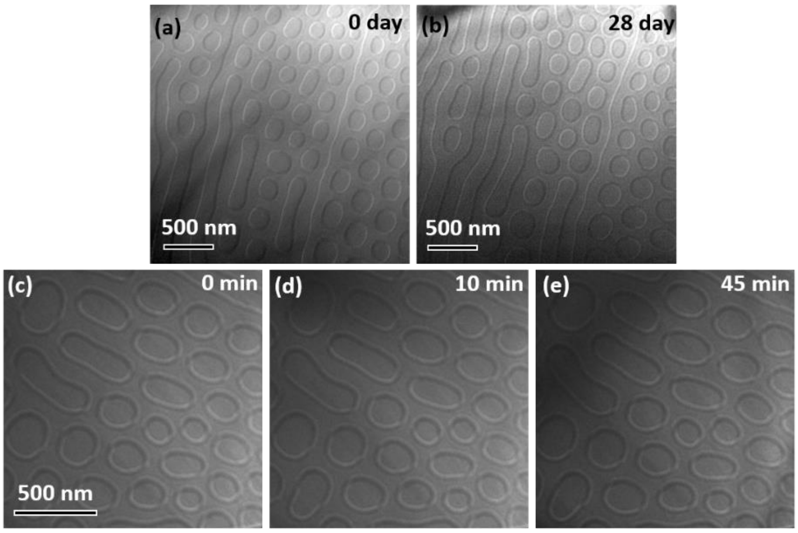

Figure S8. L-TEM images of spontaneous magnetic bubbles at room temperature. (a) Magnetic bubbles at 295 K obtained after heating the sample from 150 to 295 K at zero external field. (b) Magnetic bubbles after keeping the sample at room temperature for 28 days. (c–e) Spontaneous magnetic bubbles at 295 K after relaxing 0, 10, and 45 minutes.

In $DyFe_{11}Ti$, the labyrinth-like domains are the ground state at room temperature, but the spontaneous bubbles could remain robust at room temperature for 28 days (Figure S8a,b). The relaxation time is usually fitted as a function of temperature using the Arrhenius law, $\tau = \tau_0 \exp(E_s/k_BT)$ where τ_0 is the pre-exponential factor, k_B is the Boltzmann constant, and E_s is the activation energy.[5] The spontaneous bubbles are maintained unaltered during in-situ observation at 295 K for 45 minutes (Figure S8c–e), thus the metastable bubble state should have very long lifetime.

The zero-loss spectrum is used to measure the thickness with slit open on different spots and the mean thickness is about 100 nm by electron energy loss spectrum (EELS) (Figure

S9a) based on $t = \lambda \ln(I_t/I_0)$, where I_0 is the sum of zero-loss peak counts, I_t is the sum of total spectrum counts, and λ is the inelastic mean free path. The thickness map (Figure S9b) is measured using a slit width of 30 eV.

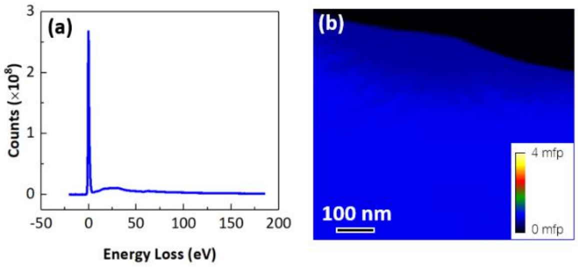

Figure S9. (a) Electron energy loss spectrum for an L-TEM specimen. The thickness t is calculated as $t = \lambda \ln(I_t/I_0)$, where I_0 is the sum of zero-loss peak counts, I_t is the sum of total spectrum counts, and λ is the inelastic mean free path. (b) Thickness map measured using a slit width of 30 eV.

References

[1] G. Asti, S. Rinaldi, *Phys. Rev. Lett.* **1972**, *28*, 1584.

[2] X. C. K. K.Yu. Guslienko, R. Grössinger, *J. Magn. Magn. Mater.* **1995**, *150*, 383-302.

[3] O. Isnard, E. J. Kinast, *Engineering* **2020**, *6*, 154.

[4] Y. Wu, J. Tang, B. Lyu, L. Kong, Y. Wang, J. Li, Y. Soh, Y. Xiong, M. Tian, H. Du, *Appl. Phys. Lett.* **2021**, *119*, 012402.

[5] L. Peng, Y. Zhang, L. Ke, T.-H. Kim, Q. Zheng, J. Yan, X.-G. Zhang, Y. Gao, S. Wang, J. Cai, B. Shen, R. J. McQueeney, A. Kaminski, M. J. Kramer, L. Zhou, *Nano Lett.* **2018**, *18*, 7777–7783.
